# Supplementary material for: Asynchronous magnetic resonance elastography: Shear wave speed reconstruction using noise correlation of incoherent waves
Source: Magn Reson Med. 2022 Oct 27;89(3):990–1001. doi: 10.1002/mrm.29502 (PMC9792433; doi:10.1002/mrm.29502)
Supplement: Supplementary file 1 — DATA S1 MATLAB code used to generate the numerical simulations of Figure 1 [file MRM-89-990-s002.zip › k-Wave/helpfiles/example_us_bmode_linear_transducer.html]

Simulating B-mode Ultrasound Images Example (k-Wave)


# Simulating B-mode Ultrasound Images Example

This example illustrates how k-Wave can be used for the simulation of B-mode ultrasound images (including tissue harmonic imaging) analogous to those produced by a modern diagnostic ultrasound scanner. It builds on the Defining An Ultrasound Transducer, Simulating Ultrasound Beam Patterns, and Using An Ultrasound Transducer As A Sensor examples.

- Open the file in the MATLAB Editor
- Run the file in MATLAB

Note, this example generates a B-mode ultrasound image from a 3D scattering phantom using `kspaceFirstOrder3D`. Compared to ray-tracing or Field II, this approach is very general. In particular, it accounts for nonlinearity, multiple scattering, power law acoustic absorption, and a finite beam width in the elevation direction. However, it is also computationally intensive. Using a modern GPU and the Parallel Computing Toolbox (with `'DataCast'` set to `'gpuArray-single'`), each scan line takes around 3 minutes to compute. Using a modern desktop CPU (with `'DataCast'` set to `'single'`), this increases to around 30 minutes. In this example, the final image is constructed using 96 scan lines. This makes the total computational time around 4.5 hours using a single GPU, or 2 days using a CPU.

To allow the simulated scan line data to be processed multiple times with different settings, the simulated RF data is saved to disk. This can be reloaded by setting `run_simulation = false` within the example m-file. The data can also be downloaded from http://www.k-wave.org/datasets/example\_us\_bmode\_scan\_lines.mat

## Contents

- Simulation approach
- Defining the scattering medium
- Running the simulation
- Processing steps
- Fundamental and harmonic imaging
- Extending the simulations

## Simulation approach

To minimise the computational overhead of running multiple 3D simulations with large grid sizes, the total number of elements in the ultrasound transducer is set to the number of active elements (in this case 32). This allows a smaller computational grid to be used. For each new scan line, the values for `medium.sound_speed` and `medium.density` within the computational grid are updated based on a larger map of the scattering phantom (see schematic below). This has the effect of simulating a larger transducer in which the active elements are swept across the transducer.

Note, the same result could be achieved by allocating the complete transducer and then progressively updating `transducer.active_elements`. However, this requires a larger computational grid for the simulation of each scan line.

## Defining the scattering medium

The contrast in an ultrasound image is generated by acoustic reflections from changes in impedance (sound speed and density). Within the human body, these heterogeneities occur across multiple length scales. At a macroscopic level, different tissue types such as fat or muscle have different bulk material properties. Specular and diffractive scattering from heterogeneities on this scale manifest as noticeable edges in the ultrasound image. At a microscopic level, there is also a difference in the acoustic properties between the cells and the extra cellular matrix in which they preside. Diffusive scattering from heterogeneities on this scale ultimately results in the speckle pattern that is characteristic of ultrasound images. (The speckle pattern is created by the constructive and destructive interference of spatial and temporal variations in these reflections. The exact speckle pattern is also strongly dependent on the characteristics of the ultrasound probe.)

To account for these different effects, the ultrasound phantom must similarly be heterogeneous across multiple scales. In this example, a scattering phantom is created by modulating the mean sound speed and density at each grid point using random Gaussian noise. To simulate bulk tissue contrast, three spherical regions with increased scattering and impedance are defined using `makeBall`. The centers of the three spheres are co-aligned with center of the transducer in the elevation direction. A schematic of the simulation layout and a slice through the scattering phantom in the beam plane are shown below.


## Running the simulation

The simulation is run in a similar way to the previous ultrasound examples. The defined transducer is used to replace both the `source` and `sensor` inputs. The scan lines are then simulated sequentially. For each scan line, the values for `medium.sound_speed` and `medium.density` within the computational domain are updated based on the larger scattering phantom. The pressure at each active transducer element (in this case 32) is then returned and stored.

```
% loop through the scan lines
for scan_line_index = 1:number_scan_lines

    % load the current section of the medium
    medium.sound_speed = sound_speed_map(:, medium_position:medium_position + Ny - 1, :);
    medium.density = density_map(:, medium_position:medium_position + Ny - 1, :);
        
    % run the simulation
    sensor_data = kspaceFirstOrder3D(kgrid, medium, transducer, transducer, input_args{:});

    % extract the scan line from the sensor data
    scan_lines(scan_line_index, :) = transducer.scan_line(sensor_data);
        
    % update medium position
    medium_position = medium_position + transducer.element_width;

end
```

## Processing steps

After simulation, a number of steps are required to process the raw transducer data into an ultrasound image. In this example, the processing follows the basic steps performed by a modern diagnostic ultrasound scanner. These are illustrated in the block diagram below. More detailed information on each of these steps can be found in any reference book on ultrasound imaging, for example, "Diagnostic Ultrasound: Principles and Instruments" by Frederick Kremkau, or "Diagnostic Ultrasound Imaging" by Thomas Szabo.

**1. Receive Beamforming**

First, the received pressure signals at each active transducer element are processed into a single scan line. This is done using the `scan_line` method of the `kWaveTransducer` class. The beamforming delays and weights are automatically calculated based on the transducer focus distance and apodization settings.

```
% extract the scan line from the sensor data
scan_lines(scan_line_index, :) = transducer.scan_line(sensor_data);
```

After beamforming, the first part of each scan line is also windowed to remove the input signal from the recorded sensor data. This step is required because k-Wave allows the transducer elements to both send and receive at the same time. (This is not physically possible using a standard ultrasound probe.)

**2. Time Gain Compensation**

The acoustic waves generated by the ultrasound transducer lose energy as they propagate through the tissue due to acoustic attenuation (absorption and scattering) and focussing effects. Consequently, reflections from deeper tissue features will appear weaker in the recorded signals. To compensate for this, a simple correction is applied assuming the attenuation within tissue follows the relation:

where alpha is the attenuation coefficient in dB/(MHz cm), f is the frequency in MHz (chosen here as the transmit frequency), and d is the total (round-trip) distance travelled in cm. Here the distance variable is created assuming that t = 0 corresponds to the middle of the transmitted tone burst.

```
% create radius variable assuming that t0 corresponds to the middle of the
% input signal
t0 = length(input_signal) * kgrid.dt / 2;
r = c0 * ( (1:length(kgrid.t_array)) * kgrid.dt / 2 - t0);    % [m]

% define absorption value and convert to correct units
tgc_alpha_db_cm = medium.alpha_coeff * (tone_burst_freq * 1e-6)^medium.alpha_power;
tgc_alpha_np_m = tgc_alpha_db_cm / 8.686 * 100;

% create time gain compensation function based on attenuation value and
% round trip distance
tgc = exp(tgc_alpha_np_m * 2 * r);

% apply the time gain compensation to each of the scan lines
scan_lines = bsxfun(@times, tgc, scan_lines);
```

**3. Frequency Filtering**

The scan lines are then filtered using a Gaussian filter centered about the transmit frequency or the second harmonic. This reduces the effects of noise outside the transmit frequency range, or in the latter case, obtains the harmonic signal for tissue harmonic imaging. The filtering is performed using `gaussianFilter` by specifying the required filter center frequency and percentage bandwidth.

```
% filter the scan lines using both the transmit frequency and the second harmonic
scan_lines_fund = gaussianFilter(scan_lines, 1/kgrid.dt, tone_burst_freq, 100, true);
scan_lines_harm = gaussianFilter(scan_lines, 1/kgrid.dt, 2 * tone_burst_freq, 30, true);
```


**4. Envelop Detection**

The high frequency variations in the scan lines are then removed in a demodulation process known as envelope detection. Here, this is achieved using `envelopeDetection` which extracts the signal envelope using the Hilbert transform.

```
% envelope detection
scan_lines_fund = envelopeDetection(scan_lines_fund);
scan_lines_harm = envelopeDetection(scan_lines_harm);
```

**5. Log Compression**

The dynamic range of the processed scan lines often exceeds the gray scale range that can be displayed by a computer monitor or visually perceived. To reduce this dynamic range, the scan lines are log compressed using `logCompression` with a normalised compression ratio of 3.

```
% normalised log compression
compression_ratio = 3;
scan_lines_fund = logCompression(scan_lines_fund, compression_ratio, true);
scan_lines_harm = logCompression(scan_lines_harm, compression_ratio, true);
```

**6. Scan Conversion**

Finally, the processed scan lines are spatially remapped to give an image resolution suitable for display. In this example, the generated image is upsampled by a factor of 2 using `interp2`.

```
% upsample the image using linear interpolation
scale_factor = 2;
scan_lines_fund = interp2(1:kgrid.Nt, (1:number_scan_lines).', scan_lines_fund, 1:kgrid.Nt, (1:1/scale_factor:number_scan_lines).');
scan_lines_harm = interp2(1:kgrid.Nt, (1:number_scan_lines).', scan_lines_harm, 1:kgrid.Nt, (1:1/scale_factor:number_scan_lines).');
```

A plot of the central scan line after each processing step is given below.


## Fundamental and harmonic imaging

The resulting B-mode images are shown below. The images look realistic and contain many of the features and artifacts seen in diagnostic images from commercial ultrasound scanners, for example, speckle and shadowing. The harmonic image has improved resolution and contrast.


## Extending the simulations

This example can be used as a template for the simulation of a wide range of B-mode ultrasound images. For instance, the scattering phantom could be replaced with one generated using 3D MRI or CT data. It is also possible to simulate the effect of different signal processing techniques, for example, frequency compounding (where the images produced using different band pass filters are averaged), spatial compounding (where the images produced when the transducer is slightly rotated are averaged), and superharmonic imaging (where the higher frequency harmonics are used to form the image).
